# Transcriptional Profiles of Drought-Related Genes in Modulating Metabolic Processes and Antioxidant Defenses in *Lolium multiflorum*

**DOI:** 10.3389/fpls.2016.00519

**Published:** 2016-04-25

**Authors:** Ling Pan, Xinquan Zhang, Jianping Wang, Xiao Ma, Meiliang Zhou, LinKai Huang, Gang Nie, Pengxi Wang, Zhongfu Yang, Ji Li

**Affiliations:** ^1^Department of Grassland Science, Sichuan Agricultural UniversityChengdu, China; ^2^Agronomy Department, University of FloridaGainesville, FL, USA; ^3^Department of Crop Molecular Breeding, Biotechnology Research Institute, Chinese Academy of Agricultural SciencesBeijing, China

**Keywords:** *Lolium multiflorum* L, differentially expressed genes, drought tolerance, metabolic processes, antioxidant defense

## Abstract

Drought is a major environmental stress that limits growth and development of cool-season annual grasses. Drought transcriptional profiles of resistant and susceptible lines were studied to understand the molecular mechanisms of drought tolerance in annual ryegrass (*Lolium multiflorum* L.). A total of 4718 genes exhibited significantly differential expression in two *L. multiflorum* lines. Additionally, up-regulated genes associated with drought response in the resistant lines were compared with susceptible lines. Gene ontology enrichment and pathway analyses revealed that genes partially encoding drought-responsive proteins as key regulators were significantly involved in carbon metabolism, lipid metabolism, and signal transduction. Comparable gene expression was used to identify the genes that contribute to the high drought tolerance in resistant lines of annual ryegrass. Moreover, we proposed the hypothesis that short-term drought have a beneficial effect on oxidation stress, which may be ascribed to a direct effect on the drought tolerance of annual ryegrass. Evidence suggests that some of the genes encoding antioxidants (HPTs, GGT, AP, 6-PGD, and G6PDH) function as antioxidant in lipid metabolism and signal transduction pathways, which have indispensable and promoting roles in drought resistance. This study provides the first transcriptome data on the induction of drought-related gene expression in annual ryegrass, especially via modulation of metabolic homeostasis, signal transduction, and antioxidant defenses to improve drought tolerance response to short-term drought stress.

## Introduction

Water availability plays a significant role in the transportation of metabolites and enzymatic reactions in plants, and also plays roles in the hydrolytic breakdown of proteins, lipids, and carbohydrates (Bewley and Black, 1994; Białecka and Kêpczyñski, 2010). Previous studies (Mittler, 2006; Liu et al., 2015) have reported that drought tolerance in plants is ascribed to the comprehensive function of multiple pathways. For example, there is a general increase in metabolite levels under drought stress, including amino acids, sugars, and alcohols (Witt et al., 2012). Similarly, drought tolerant plants have reaction mechanisms to maintain cellular homeostasis by lipid metabolism and can regulate metabolic homeostasis (Da Silva et al., 1974). The metabolite changes contribute to the number of drought-responsive genes when the plant is under abiotic stress. The genes that encode structural proteins and regulatory proteins involved in metabolic pathways have been shown to be strongly associated with drought tolerance (Song et al., 2005; Xiao et al., 2006). Actually, a number of genes are known to be related to early response to stress, and are thought to be involved in metabolic processes possessing adaptation capacity to abiotic stress (Puranik et al., 2011). The identification such genes has been suggested as representing a promising approach toward the improvement of drought tolerance in crops (Munns, 2005). Responsive genes have been identified through high-throughput sequencing technology, which is a powerful method that is used to analyze changes in cell morphology, gene expression, and physiological and biochemical metabolism for plants under abiotic and biotic stress (Lan et al., 2012).

Drought stress will inevitably result in oxidative damage in plants due to over production of reactive oxygen species (ROS), which are highly reactive and toxic. High ROS level not only cause damage to proteins, lipids, and carbohydrates, but also acts as signal molecules (Petrov et al., 2015). It was recently reported that elevated antioxidant levels are associated with plant tolerance to abiotic stressors (Sudhakar et al., 2001). For example, Mn-SOD, an important antioxidant enzyme, may play a role in the drought tolerance of rice (Wang et al., 2005). The overexpression of ascorbate peroxidase (APX) in *Nicotiana tabacum* chloroplasts was shown to enhance salt and drought tolerance in the species (Badawi et al., 2004a). Similarly, the overexpression of monodehydroascorbate reductase (MDAR) in transgenic tobacco can increase salt tolerance (Badawi et al., 2004b). Additionally, the overexpression of glutathione S-transferases (GST) and dehydroascorbate reductase (DHAR) enhances plant tolerance to various abiotic stressors (Eltayeb et al., 2007). These results suggest that genes encoding antioxidant enzymes are essential for improving drought tolerance across several plant genera. Therefore, to study antioxidant systems has an important meaning on displaying the mechanism of stress resistance for the plants subjected to abiotic stress.

Drought is a common environmental stress on annual grass productivity in low rainfall areas around the world. The decrease of annual precipitation has become the most critical factor that effects annual ryegrass (*Lolium multiflorum* L.) germination and establishment (Wang et al., 2005). Annual ryegrass is an important short-duration, cool-season grass that is widely distributed among the world. In southern China, it is most widely used as an annual forage crop and has an increasing area for forage production (Zhang et al., 2008). Although annual ryegrass is a drought resistant grass and has a high root growth capacity, drought still causes a dramatic reduction of tillers and leaves (Antolin et al., 1995).

There is little information concerning the identification of drought-related genes in annual ryegrass. In this study we explored antioxidant–related genes that may contribute to drought tolerance in annual ryegrass. Here we report the results of the regulatory mechanism of annual ryegrass in response to short-term drought stress. The identification of drought-related genes has a vital role in the modulation of metabolic processes and development of antioxidant defenses to improve drought tolerance of annual ryegrass. This study aimed to provide candidate genes to facilitate the genetic improvement of annual ryegrass for its use as a sustainable forage crop.

## Materials and methods

### Plant materials and drought stress treatments

Seeds of two *L. multiflorum* lines, “Abundant 10 (drought-resistant)” and “Adrenalin 11 (drought susceptible),” were germinated at 25°C on filter paper that was wetted with distilled water. After seven days of growth, seedlings were transplanted into plastic pots (5 × 5 cm) filled with the Hoagland's nutrient solution in temperature-controlled growth chambers. The environmental conditions were as follows: temperature 25°C during the day and 18°C at night, photon flux density 900 umolm^−2^ s^−1^, a photoperiod of 16/8 h for the day/night cycle, and a relative humidity of 60%.

To study the effects of short-term drought stress on the physiological response of two *L. multiflorum* lines, the 20-day-old seedlings were split into two halves. One half of the seedlings were used as the control, and the other half of seedlings were naturally air dried (drought treatment) for 1 and 2 h, respectively. The drought-stressed and well-watered seedlings were immediately frozen in liquid nitrogen for RNA-Seq. The leaf transpiration rate was determined by a gravimetric method (Aroca et al., 2003). Leaf water potential was calculated with the formula described by Boyer (1970). Hydrogen peroxide content (H_2_O_2_), Malondialdehyde (MDA) concentration, superoxide dismutase (SOD), catalase (CAT), dehydroascorbate reductase (DHAR), and monodehydroasorbate reductase (MDHAR) activities, glucose and fructose concentrations were assayed separately with hydrogen peroxide, MDA, SOD, CAT, DHAP, MDHAR, glucose, and fructose assay kits (Comin Biotechnology Co., Ltd. Suzhou, China). Statistical analysis was performed by one-way ANOVA using SPSS Statistics 20.0. Least Significant Difference (LSD) values were calculated when specific parameters changed significantly (*P* < 0.05).

### RNA extraction and library preparation

Ten individual plants were pooled to create one treatment, and two biological replicates were used for all RNA-Seq analysis for each treatment. Total RNA of each sample was extracted using the Trizol reagent (Agilent RNA 6000 nano Reagents) according to the manufacturer's instructions. The total RNA concentration, RNA integrity number (RIN), and 28S/18S were detected using an Agilent 2100 Bioanalyer (Agilent RNA 6000 Nano Kit). The purity of the samples was tested using a NanoDrop. After RNA isolation and quality assessment, samples were stored at −80°C until the cDNA library construction and transcriptomic assay were completed. Only those samples with an RIN between 6 and 7 and a 28S/18 S ratio within the range of 1.5–2 were qualified for cDNA library preparation.

### Library construction and sequencing

A total of 5 ug of total RNA per sample was used to construct the cDNA library using the NEB Next Ultra RNA Library Prep Kit for Illumina (New England Biol-abs (NEB), USA). Initially, the total RNA sample was digested using Dnase I (NEB) and purified by oligo-dT beads (Dynabeads mRNA purification kit, Invitrogen). Following purification, the poly (A)-containing mRNA was fragmented into 200–250 bp pieces using fragment buffer (Ambion), and the first-strand cDNA was synthesized using the N6 primer, First Strand Master Mix and Super Script II reverse transcription (Invitrogen). The reaction conditions were as follows: 25°C for 10 min; 42°C for 30 min; 70°C for 15 min; 4°C Hold. We then added a Second Strand Master Mix to generate the second strand cDNA (16°C for 2 h). The cDNA fragments were purified using a QIAquick PCR Purification Kit (QIAGEN), and these cDNA fragments were combined with an End Repair Mix (20°C for 30 min). Subsequently, the cDNA fragments were purified, the A-Tailing Mix was added, and the mixture was incubated for the ligation reaction at 20°C for 20 min. The products were then run on a 2% agarose gel to select fragments within 300–350 bp. The gel was purified with a QIAquick Gel Extraction kit (QIAGEN). Several rounds of PCR amplification with a PCR Primer Cocktail and a PCR Master Mix were performed to enrich the cDNA fragments. The PCR products were then purified using Ampure XP Beads (AGENCOURT). The cDNA library was validated by two different methods to determine the average molecular length using the Agilent 2100 bioanalyzer instrument (Agilent DNA 1000 Reagents), and by real-time quantitative PCR (QPCR; TaqMan Probe). The qualified libraries were amplified on cBot to generate a cluster on the flowcell (TruSeq PE Cluster Kit V3-cBot-HS, Illumina), and the amplified flowcell were paired- end sequenced on the HiSeq 2000 System (TruSeq SBS KIT-HS V3, Illumina).

### Transcriptome analysis

Raw reads from twelve libraries were generated and were transformed from image data output into sequence data. Sequencing quality value (SQ) of the sequence data was in the range of 2–35. Raw reads with only adapters and unknown or low quality bases, were removed, and the following analyses were based on clean reads only. *De novo* assembly of RNA-seq was conducted using Trinity (http://trinityrnaseq.sourceforge.net/). The unigenes were generated by Trinity modules and the processes of sequence splicing and redundancy removing were employed. The unigenes were divided into two classes: clusters and singletons. Clusters (CL) are group of unigenes with a sequence similarity of greater than 70% among them. Distinct clustering was performed to cluster the assembled unigenes transcript sequences to identify the same gene or homolog using a hierarchical clustering approach involving TGICL-CAP3 and CD-HIT20. The alignment results were used to decide the sequence direction of the unigenes using TGICL 2.1 (http://sourceforge.net/projects/tgicl/files/tgicl%20v2.1/) and Phrap Release 23.0 (http://www.phrap.org/). If the results of the databases conflicted with each other, a priority order of NR, Swiss-Prot, KEGG, and COG was followed when deciding the sequence direction of unigenes. When a unigene was unaligned in any of the above databases the ESTS software was used to decide the sequence direction from the 5′ end to the 3′ end.

### Differential expression and pathway analysis

The fragments per kb per million fragments (FPKM) of each unigene in each library were calculated using Cufflinks (http://cufflinks.cbcb.umd.edu/). Referring to Audic and Claverie (1997), we used a rigorous algorithm to identify differentially expressed genes (DEGs) between two samples, and the False Discovery Rate (FDR) as a statistical method in multiple hypotheses testing to correct for *p*-value to guarantee the low FDR. A very stringent cutoff, FDR ≤ 0.001 and a fold change value of 2, were used to identify DEGs. Tool edgeR23 was used to identify significantly up- and down-regulated genes on the read count values of genes.

A GO functional analysis and KEGG Pathway analysis were conducted on DEGs. First, all of the DEGs were BLASTed in the GO database (http://www.geneontology.org/) and the gene numbers were calculated for each GO term with GO-Term Finder v. 0.86 (http://search.cpan.org/dist/GO-TermFinder/). Then, a hyper geometric test was used to find significantly enriched GO terms in DEGs to compare with the genome background using the *p*-value. The calculated *p*-value was calibrated using the Bonferroni correction. GO terms were defined as significantly enriched GO terms in DEGs, if corrected *p* ≤ 0.05. Pathway enrichment analysis identifies significantly enriched metabolic pathways or signal transduction pathways in DEGs when compared with the whole genome background. The calculated *p*-value for the pathway enrichment analysis was similar to that in the GO analysis. After multiple testing corrections, the pathways with a *p* ≤ 0.05 were considered significantly enriched in DEGs.

### Quantitative real-time-PCR analysis

To validate the RNA-seq data, 14 genes were randomly selected to be analyzed by qRT-PCR with a reference gene (Actin; Table 1). Sample treatment and RNA isolation were obtained following previously described above. Three independent biological replicates of each sample and three technical replicates of each biological replicate were used in the RT-PCR using the ABI7500 Fast Real-Time PCR System (Applied Bio-systems, USA) with a 96-well block. The reverse-transcription reactions were performed using the iScript™ advanced cDNA Synthesis Kit (BIO-RAD). PCR amplifications were performed in 20 uL total volume reactions containing 2.5 uL templates, 10 uL reaction Mix, 5.5 uL ddH_2_O, and 1 uL of each primer. The reaction conditions were 30 s at 95°C, followed by 40 cycles of 95°C for 5 s and 60°C for 34 s. The melting-curve was obtained by applying increasing temperature from 58 to 95°C. To determine the relative fold change for each sample in each experiment the CT-value for the reference gene and six randomly chosen genes were calculated using the CT method as previously described (Livak and Schmittgen, 2001).

**Table 1:** **Primer sequences used for Quantitative real-time-PCR analysis**.

**Seq-ID**	**Gene name**	**Primer Sequence (5′-3′) and Tm(°C)**	**GO-Cellular Component**
AJ585201	Actin	F TCCTCACGCCATTCTT (59.10) R TCTCCTTGATGTCCCT (59.08)	GO:0008372; cellular component
Unigene42814	Sterol 24-C-methyltransferase	F CGAACTTCAAGCACACTCGT (59.09) R CAGTACCCTTTGGTGCAATG (59.05)	GO:0009506//plasmodesma;GO:0005773//vacuole;GO:0005783//endoplasmic reticulum
Unigene42248	GDP-D-mannose 3′, 5′-epimerase	F AATCCAACCATTCGGTCATT (59.10) R TACAAGCCGATGAGGCATAG (58.90)	–
Unigene19563	Acyl-CoA dehydrogenase	F GCAACCAACATTGAAACGAG (59.17) R TGATGACGAGGCTTAGTTGG (58.87)	–
CL1489	Predicted protein	F GCTATTACCGCAAGGTCCAT (59.07) R TCGATCATATGGCACAACAA (58.49)	–
CL11729	Abscisic acid receptor PYR/PYL family	F GCTGCACTTCACCAAAGAAA (59.05) R TGCTCTCGAATTTCTCAACG (59.15)	–
CL10698	Hexokinase	F TGGACCTAGGAGGGACAAAC (58.99) R AACCAAACAATTCCGAGGAG (59.03)	GO:0005739//mitochondrion;GO:0009536//plastid;GO:0005773//vacuole;GO:0005634//nucleus;GO:0005886//plasma membrane
Unigene30003	Hypothetical protein CHLNCDRAFT_56419	F TCCGCAAACAAATCGTAGAG (58.92) R CAAAGACAGGACCAGCAGAA (59.01)	–
CL12576	3β-hydroxysteroid-dehydrogenase	F AAGCATACACGCAAACGAAG (58.99) R CAAGTGCGCAGCTCATTTAT (59.09)	GO:0016020//membrane
Unigene48238	Uncharacterized protein LOC100501669	F GAAGAGGTATGCTGATGGCA (58.83) R AGACCATACGCCTTGGTAGC (59.22)	GO:0005739//mitochondrion;GO:0009579//thylakoid;GO:0009507//chloroplast;GO:0016020//membrane;GO:0005783//endoplasmic reticulum
Unigene2564	Hypothetical protein PHYSODRAFT_484706	F GCCGATCCAACTCATACCTT (59.01) R TGCATGCTTGAGGATACCAT (59.10)	GO:0009941//chloroplast velope;O:0048046//apoplast;GO:0010319//stromule;GO:0005829//cytosol;GO:0009570//chloroplaststroma;GO:0009579//thylakoid
Unigene3201	Shaggy-related protein kinase	F CTCGTAACACCGACACCATC (59.01) R CGTTGTCTAACATCCATCGG (59.00)	GO:0005829//cytosol
Unigene28957	Predicted protein	F ACGACCAAAGTGGTGAACAA (59.03) R TGCCGAAATCAAGACACAAT (59.13)	GO:0009941//chloroplast envelope;GO:0005794//Golgi apparatus;GO:0005783//endoplasmic reticulum
Unigene41363	Predicted protein	F CGACTACAAGGACTGGCAGA (59.03) R CAATACCACCCACAGCTTCA (59.57)	–
Unigene21403	Aspartate aminotransferase	F GGGATGCATTTGGAGATGA (59.39) R TGAGCAGAGTCGTTGCTTCT (58.91)	GO:0009507//chloroplast
CL7857	Os08g0127100	F TTCAGCCTCTCATGGTTCAC (58.80) R GCCACGATCATCAGAATCAC (59.04)	GO:0016021//integral to membrane; GO:0005886//plasma membrane

## Results

### Drought-induced antioxidant enzymes in two *L. multiflorum* lines and their responses to drought stress

**Figure 1:**
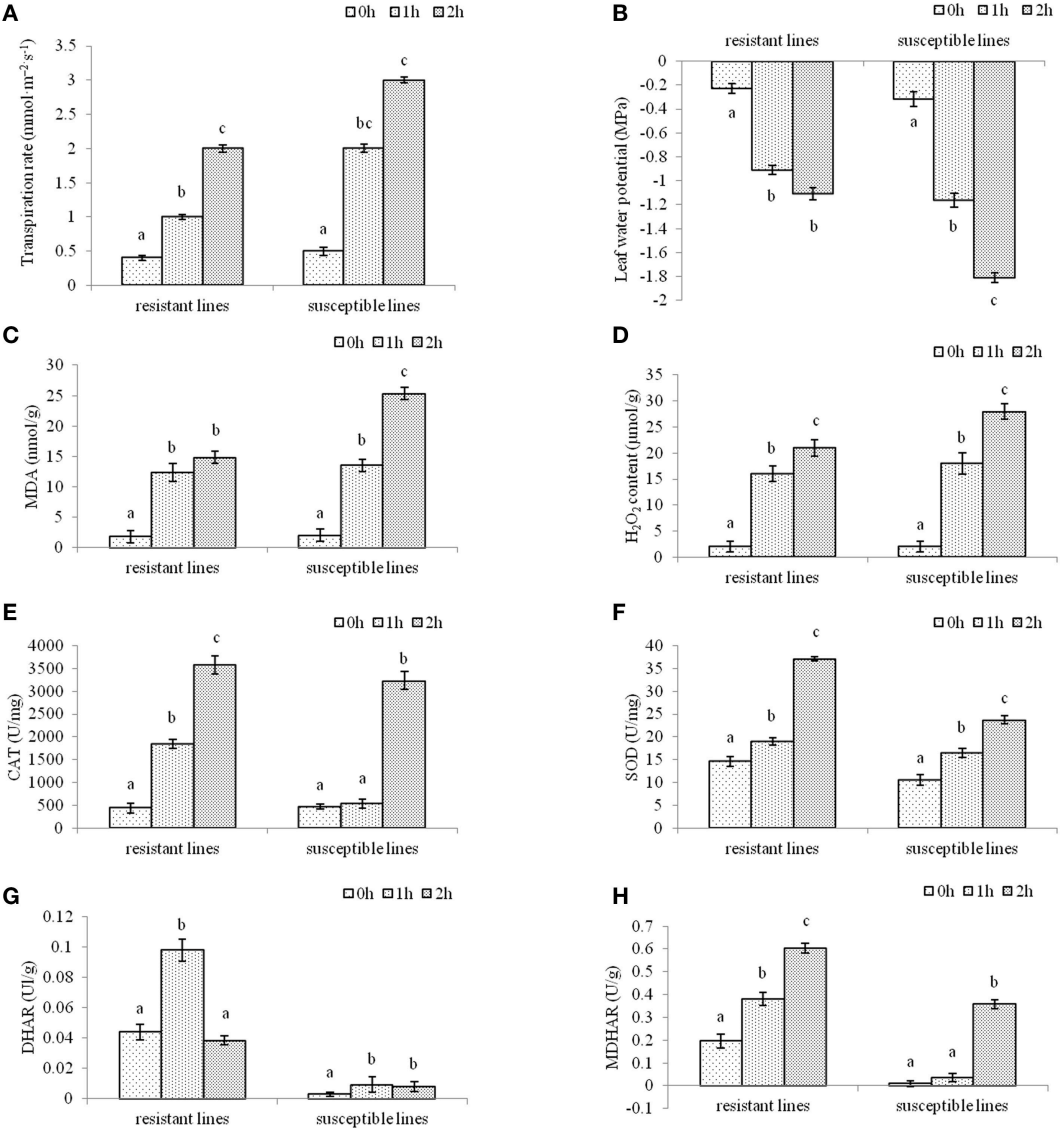
**The physiological indexes of ***L. multiflorum*** were measured after drought treatments**. Leaf transpiration rate **(A)** and leaf water potential **(B)** in drought-resistant and drought-susceptible plants after 0, 1, and 2 h of treatment **(A,B)**. Malondialdehvde content **(C)** and H_2_O_2_ concentration **(D)** of 20-day-old annual ryegrass lines with drought stress for 0, 1, and 2 h **(C,D)**. The enzymatic activities of catalases **(E)**, superoxide dismutase **(F)**, dehydroascorbate reductase **(G)**, and monodehydroasorbate reductase **(H)** of two *L. multiflorum* lines after different drought treatments **(E–G)**. Bars with different letters above the columns of figures indicate significant differences (*P* < 0.05) between different time points.

To investigate the effects of drought stress resulting in oxidative stress in two *L. multiflorum* lines, enzyme change was quantified after plants were treated with drought stress for 1 or 2 h. The results showed that there was a significant difference in non-treated and drought-treated seedlings. Leaf transpiration rate and leaf water potential in two *L. multiflorum* lines after 1 and 2 h of treatment had significant difference compared with control, especially for susceptible lines (Figures 1A,B). As a major indicator of the stress-triggered ROS level oxidative damage (Badawi et al., 2004b; He et al., 2012), malondialdehvde (MDA) content was measured in well-watered and drought-treated plants. There were significant differences among the treated and control seedlings (Figure 1C). A lower H_2_O_2_ concentration in drought-tolerant lines could be due to the higher activity of the enzymatic activities of catalases (CAT), superoxide dismutase (SOD), dehydroascorbate reductase (DHAR), and monodehydroasorbate reductase (MDHAR; Figures 1D–H). These results indicated that ROS accumulation was increased by the development of an antioxidant defense system in two *L. multiflorum* lines exposed to a short-term drought. The resistant lines faced less oxidative damage than the susceptible lines.

### Read generation and *De novo* assembly

The 12 RNA samples from two *L. multiflorum* lines were sequenced using the Illumina HiSeq 2000 platform, which generated 0.4 billion clean reads. After filtering for quality, a total of 39.8 million clean reads were assembled into 128,622 unigenes having an average length of 870 nt. Over 90% of the cleaned reads were able to be mapped to the assembly (Table 2; Accession nos: GSE78738 in the NCBI GEO database). In total, 600, 565 consensus sequences were generated after clustering the similar sequences with greater than 70% sequence similarity.

**Table 2:** **The numbers of mapped reads and assembled unigenes**.

**Samples**	**Sequenced reads**	**Mapping ratio (%)**	**Total consensus sequences**	**Distinct clusters**	**Distinct singetons**
Resistant-0	73,659,506	92.0	105,900	35,928	69,972
Reisitant-1	71,287,722	91.8	120,531	34,640	85,891
Resistant-2	73,094,738	91.3	98,840	32,319	66,521
Susceptible-0	70,721,532	92.0	96,611	31,201	65,410
Susceptible-1	72,176,332	91.9	85,628	24,417	58,211
Susceptible-2	72,072,732	92.3	93,055	28,477	64,608
Total	433,012,562		600,565	53,829	410,613

*Total Consensus Sequences represents all the assembled unigenes; Distinct Clusters represents the cluster unigenes; The same cluster contains some highly similar (more than 70%) unigenes, and these unigenes may come from same gene or homologous gene; Distinct Singletons means that this unigene come from a single gene. The number 1 and 2 represent drought treatment time for 1 and 2 h, respectively*.

### Differential expression and GO enrichment analysis

**Figure 2:**
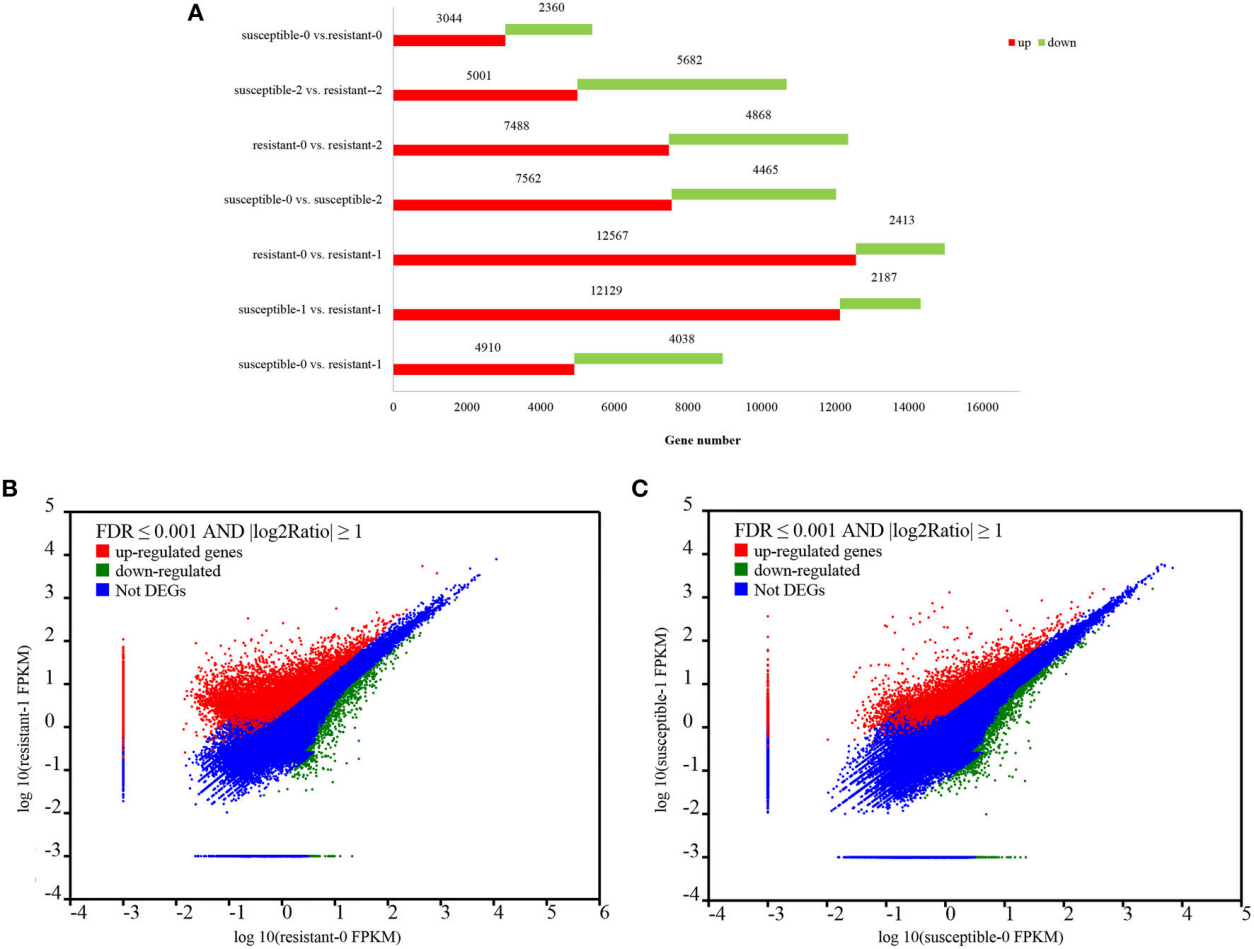
**Statistics of up- and down-regulated genes between the two ***L. multiflorum*** lines (A); The expression level of DEGs in resistant and susceptible lines exposed to drought (B–C)**. The upper with red regions reveal those genes with significantly up-regulated expression. Similarly, the lower with green dots shows the down-regulated genes and the blue dots represent the region with no DGEs.

To analyze drought-mediated gene expression of two *L. multiflorum* lines, a differential expression analysis was performed on resistant and susceptible lines. A total of 78,754 genes were significantly changed by treating plants with drought for 1 and 2 h, of which 52,721 were up–regulated genes and 26,033 were down-regulated genes (Figure 2A), Specific information regarding up-regulated and down-regulated genes is listed in Supplementary Table 1. Moreover, a total of 4718 DEGs were identified by using NOlSeq analysis (|logFC|> 1, Probability > 0.7; Tarazona et al., 2011; Supplementary Figure 1). More genes were significantly up-regulated in the resistant lines when compared with the susceptible lines under short-term drought stress in Figures 2B,C).

**Figure 3:**
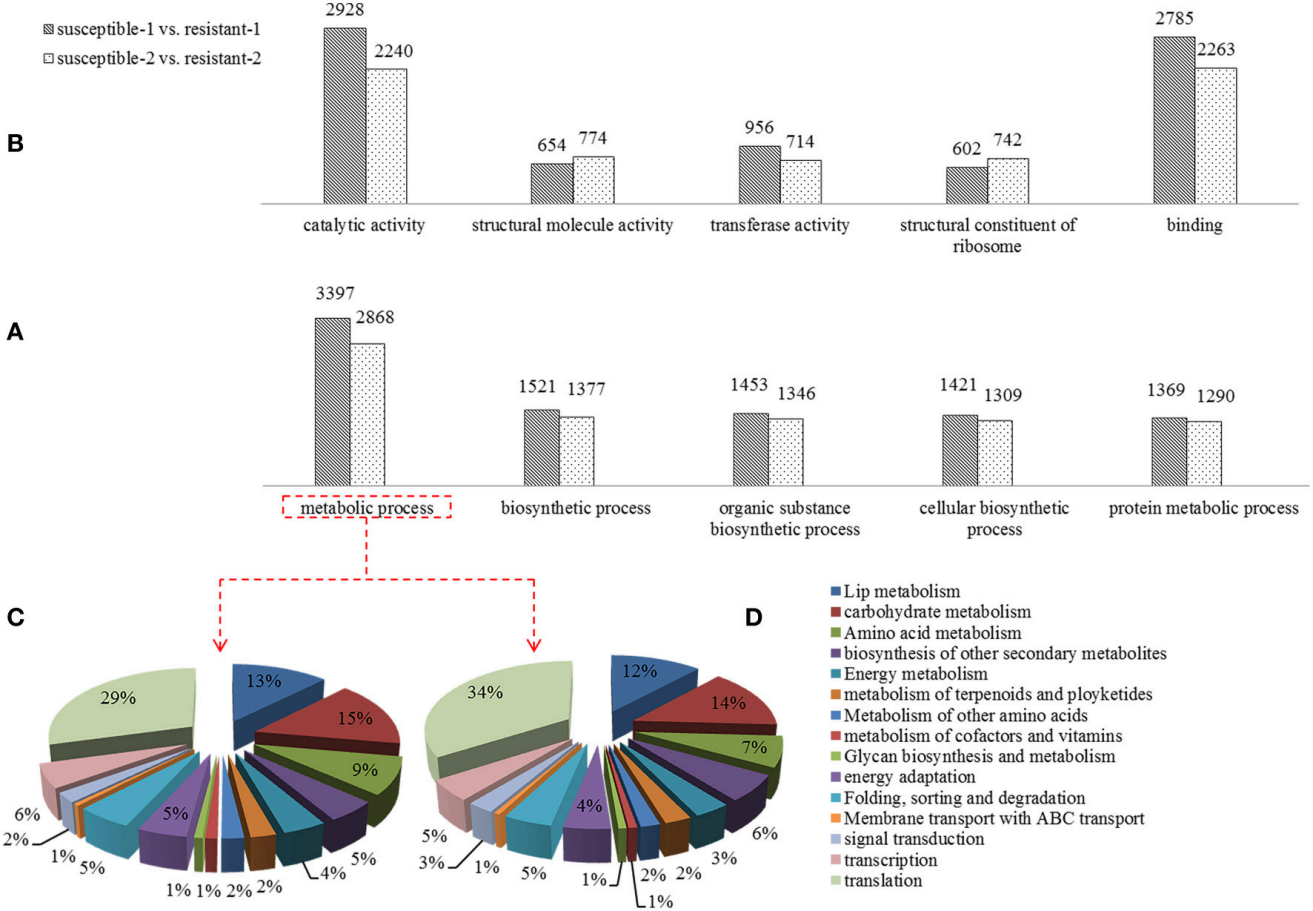
**Comparison of differentially expressed genes of the Gene Ontology (GO) analysis including biological process (A) and molecular functions (B) in two annual ryegrass lines subjected to drought stress for 1 and 2 h**. And the percent of DEGs involved in metabolic processes in resistant and susceptible lines is shown in **(C,D)**.

A Gene Ontology (GO) analysis was conducted to reveal the biological processes of DEGs that were exposed to drought stress. A large number of DEGs related to metabolic processes such as carbohydrate metabolism, lip metabolism and signal transduction were identified (Figure 3A). The percentage of DGEs was used to evaluate the importance of these metabolic processes, suggesting that lipid metabolism, carbohydrate metabolism, and signal transduction are dramatically affected by drought (Figures 3C,D). The pathways that are known to be involved in regulating mechanisms were differentially expressed under drought. Furthermore, the different functional characteristics of DEGs such as catalytic activity, transferase activity, binding, and structural constituent of the ribosome were discovered (Figure 3B).

**Figure 4:**
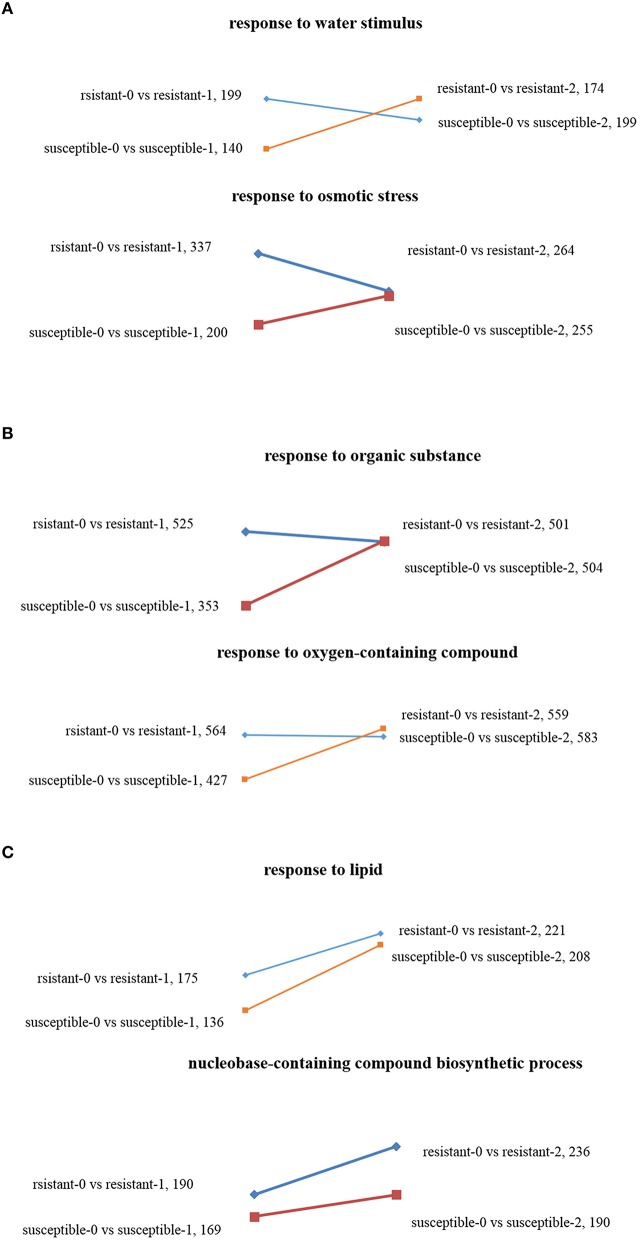
**Scatter diagrams of different changing trends of DEGs in two ***L. multiflorum*** lines by GO enrichment analysis after drought treatment for 1 and 2 h**. The GO terms included response to osmotic stress and response to a water stimulus **(A)**, response to an organic substance and response to an oxygen-containing compound **(B)**, and response to lipid and nucleobase-containing compound biosynthetic process **(C)**.

Using GO enrichment analysis, the changes of transcript abundance of enriched genes were observed by comparing plants treated with drought 1 and 2 h. For resistant lines, the number of enriched genes significantly decreased in terms of “response to osmotic stress” and “response to a water stimulus,” whereas the opposite result was observed in susceptible lines (Figure 4A). The DEGs of “response to an organic substance” and “response to an oxygen-containing compound” were not significantly enriched in tolerant lines, but were dramatically enriched in susceptible lines (Figure 4B). The numbers of DEGs found in “response to lipids” and “nucleobase-containing compound biosynthetic process” increased in the two *L. multiflorum* lines when treated with 1–2 h of drought stress (Figure 4C).

### Quantitative real-time-PCR validation of differentially expressed transcripts from RNA-Seq

**Figure 5:**
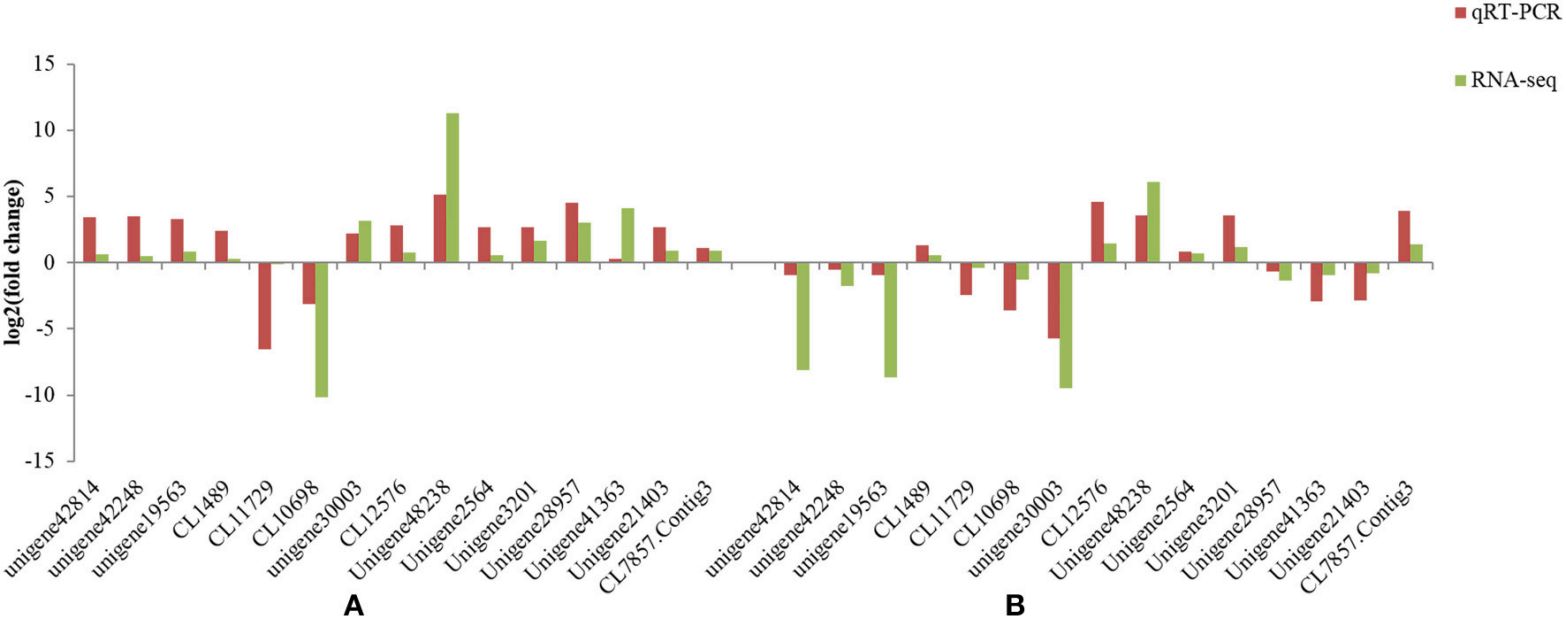
**Comparison of the different expression trends among 15 genes using both qRT-PCR results and RNA-Seq data in the resistant lines (A) and susceptible lines (B) of ***L. multiflorum*****.

To confirm the RNA-Seq results, select genes that showed up- and down-regulation in the two *L. multiflorum* lines were chosen for qRT-PCR. The relative gene expression of qRT-PCR was calculated using the 2^−ΔΔCt^ method. The expression trends of these genes agreed with the RNA-Seq data (Figure 5), and a significant correlation of fold change was identified in resistant lines (*r*^2^ = 0.982) and susceptible lines (*r*^2^ = 0.814), suggesting that reliable RNA-Seq results were obtained in this study.

### Responses of carbon metabolism-associated genes to drought stress

**Figure 6:**
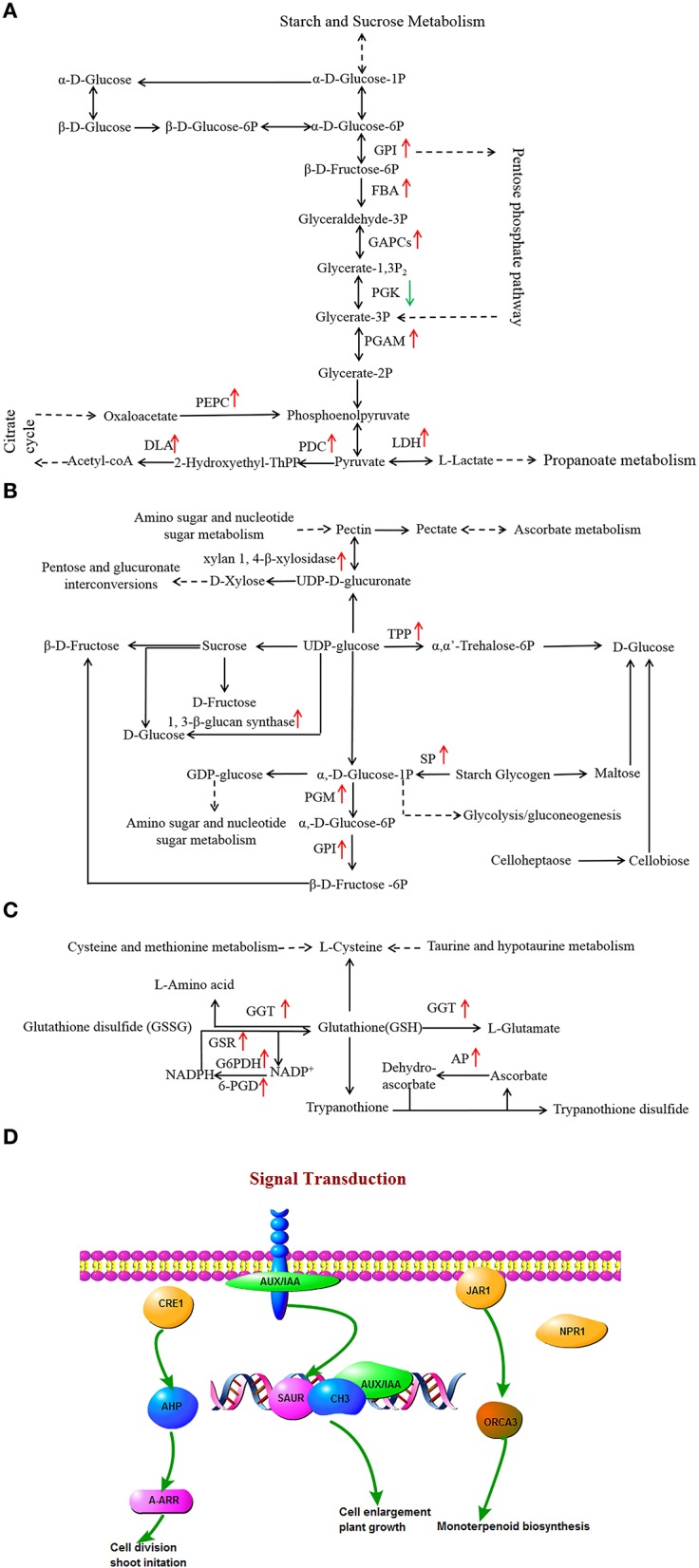
**Effects of drought stress on the expression of genes associated with glycolysis and gluconeogenesis (A), starch and sucrose metabolism (B), glycerophospholipid metabolism (C), and signal transduction (D) in resistant lines**. The red arrow indicates significantly up-regulated expression.

Glycolysis and gluconeogenesis pathways are primary metabolism pathways that may be modulated to establish a new homeostasis under drought stress in plants (Caruso et al., 2009). Changes in the expression levels of a large number of glycolysis and gluconeogenesis-associated genes in two *L. multiflorum* lines were observed (Figure 6A). Genes encoding enzymes, specifically glucose-6-phosphate isomerase (GPI) and probable phosphoglycerate mutase (PGAM) showed significant up-regulation in the resistant lines, whereas no change was observed in the susceptible lines. L-lactate dehydrogenase (LDH) participated in up-regulation in resistant lines but was down-regulated in susceptible lines. A contrasting result was observed with phosphoglycerate kinase (PGK), which was down-regulated in resistant lines and up-regulated in susceptible lines. In addition, a large proportion of the genes encoding enzymes, fructose-bisphosphate aldolase (FBA), glyceraldehyde 3-phosphate dehydrogenase (GAPCs), pyruvate decarboxylase (PDC), dihydrolipoamide acetyltransferase (DLA), phosphoenolpyruvate carboxykinase (PEPC) showed up-regulated expression in the resistant lines. Drought induced a relatively large increase in the levels of glucose in resistant lines and resulted in a sharp increase in fructose, especially when plants were treated with drought stress for 1 h, as shown in Supplementary Figures 2A,B. These results indicated that the activities of glycolysis and gluconeogenesis-associated genes may play a role in the response of resistant lines to drought.

Changes in the expression of several starch and sucrose metabolic genes in resistant lines were observed (Figure 6B). There were two genes annotated as encoding trehalose 6-phosphate synthase/phosphatase (TPP), and xylan 1, 4-β-xylosidase, which are both involved in starch and sucrose metabolism, and which showed up-regulation in resistant lines and down-regulation in susceptible lines. The 1, 3-β-glucan synthase, phosphoglucomutase (PGM), GPI, and starch phosphorylase (SP) participated in up-regulation in resistant lines, whereas no changes were observed in the susceptible lines.

### Lipid metabolism related to gene expression response to drought stress

A decrease in membrane lipid content was caused by the degradative processes that are induced by drought (De Paula et al., 1990). Lipids are important membrane components and the change in their composition may help to maintain membrane integrity and preserve cell compartmentation under water stress conditions. In response to drought stress, the lipid content in leaves decreased and fatty acids represented 61% of contents in *Arabidopsis thaliana* (Gigon et al., 2004). Several DEGs related to glycerophospholipid metabolism have significant expression changes in the two *L. multiflorum* lines (Figure 6C). Genes related to gamma-glutamyltranspeptidase (GGT), glutathione reductase (GSR), L-ascorbate peroxidase (AP), and 6-phosphogluconate dehydrogenase (6-PGD) showed up-regulated expression in resistant lines, whereas these enzymes exhibited down-regulated expression in susceptible lines. The gene encoding enzyme glucose-6-phosphate 1-dehydrogenase (G6PDH) was obviously up-regulated in resistant lines, while the enzyme was not significantly up-or down-regulated in glycerophospholipid metabolism in the susceptible lines. For ether lipid metabolism, 1-alkyl-2-acetylglycerophosphocholine esterase was affected in the resistant lines but no changes were observed in the susceptible lines.

### Signal transduction-related genes in two *L. multiflorum* lines

Gene encoding hormone signal transduction enzymes may participate in several critical processes of plant morphogenesis such as cell division and enlargement, stem growth and stomatal closure (del Pozo and Ramirez-Parra, 2015). Under drought stress, a number of DEGs encoding transcriptional factors were identified in resistant lines (Figure 6D). Among them, 1-phosphatidylinositol-4-phosphate 5-kinase (EC 2.7.1.68), phosphatidylinositol phospholipase C (EC 3.1.4.11), 1D-myo-Inositol-tetrakisphosphate 5-kinase (EC 2.7.1.140), diacylglycerol kinase (DGK; EC 2.7.1.107), and 1-phosphatidylinositol-4-phosphate 5-kinase (EC 2.7.1.68) exhibited up-regulation in the resistant lines. The remaining DEG encoded phosphatidate cytidylyltransferase (EC 2.7.7.41), which was observed as down-regulated under drought stress. Furthermore, the LAX family gene, auxin influx carrier (AUX1), was up-regulated in resistant lines and down-regulated in the susceptible lines. In contrast, three of the drought-responsive proteins (jasmonic acid-amino synthetase, histidine-containing phosphotransfer protein, and regulatory protein NPR1) were observed up-regulated only in the resistant lines.

## Discussion

### The response of drought-induced antioxidant enzymes to oxidative stress

Oxidative damage remains a potential problem as it interrupts normal plant metabolism. The level of physiological response depends on the species, the stage of development, and the metabolic state of the plant, as well as the duration and intensity of the stress (Pastori et al., 2000). ROS are produced in plants under various stress conditions and serve as important mediators in plant responses to stresses. Oxidation stress has been found to enhance drought tolerance of plant species (Zhu, 2001). However, little is known about the genes that encode antioxidant involved in drought tolerance and antioxidants defenses in annual ryegrass to short-term drought conditions. Superoxide dismutase (SOD), dehydroascorbate reductase (DHAR), and monodehydroasorbate reductase (MDHAR) are the most effective intracellular enzymatic antioxidant, and they are important in plant stress tolerance by providing defense against the toxic effects of elevated levels of ROS (Gill and Tuteja, 2010). Catalases (CAT) are indispensable for the detoxification of ROS in stress inducing environments (Garg and Manchanda, 2009). In this study, resistant lines showed higher activity of the enzymatic activities of CAT, DHAR, and MDHAR than susceptible lines in both two time points subjected to high H_2_O_2_ content. But drought had no significant effect on activity of SOD and MAD content with drought for 1 h. These results indicated that most of activities of antioxidant enzymes significantly increased to maintain cellular ROS for osmotic adaptation in drought resistant lines. Based on the result of the transcriptome, several genes encoding histidine-containing phosphotransfer proteins (HPTs), GGT, L-ascorbate peroxidase (AP), 6-phosphogluconate dehydrogenase (6-PGD), and G6PDH function as antioxidants and play an important role in increasing the tolerance of resistant lines against drought stress.

### Carbon metabolism-related genes contributed to enhanced drought tolerance

A change in glycolysis and gluconeogenesis is considered to be a basic characteristic of plant adaption to abiotic stress. Glycolysis is an important metabolic pathway in carbohydrate metabolism, and drought stress leads to altered sucrose and amino acid contents, which was revealed by metabolite analysis (Broeckling et al., 2005). Genes encoding enzymes showed significant regulation in glycolysis and gluconeogenesis metabolism and the results are consistent with a drought-mediated function in photosynthetic carbon metabolism (Kovács et al., 2006; Shaar-Moshe et al., 2015). In our study, several key enzymes in glycolysis/gluconeogenesis metabolism were differentially expressed in resistant and susceptible lines under drought stress. Genes encoding hexokinase (HxK) showed up-regulation in resistant lines. The activity of HxK is expected to be critical to the cellular levels of glucose and fructose, and the reactions catalyzed by HxK lead to hexoses entering the glycolytic pathway. In cereal crops, it has been shown that HxK decreased at the transcript levels in wheat and rice under drought stress (Xue et al., 2008; Lenka et al., 2011), but in maize HXK was found to be up-regulated under drought-stress after recovery irrigation (Hayano-Kanashiro et al., 2009). Moreover, putative phosphoglycerate mutase (PGAM) as a potential target is regulated by an identified miRNA in the glycolysis pathway (Guzman et al., 2012). The abundance of PGAM decreased under water deficit (Shu et al., 2011; Cramer et al., 2013). In our study, PGAM was significantly up-regulated in the resistant lines, suggesting that the protein might contribute to enhanced drought tolerance. L-lactate dehydrogenase (LDH) belongs to one of the most intensely studied enzyme families. L-lactate dehydrogenase is a hydrogen transfer enzyme that catalyzes the reversible reaction of pyruvate to lactate, with nicotinamide-adenine dinucleotide (NADH) as the hydrogen donor. Genes encoding LDH were up-regulated in the resistant lines of annual ryegrass. This is consistent with the results obtained through oxidative stress studies in *Saccharomyces cerevisiae* (Zhao et al., 2015). FBA is a constituent of both the glycolysis/gluconeogenesis pathway and the pentose phosphate cycle in plants (Konishi et al., 2004). FBA was up-regulated in the drought resistant lines, which validates the findings of Gong et al. (2010), who detected FBA in drought tolerant lines of tomato. It is interesting that we identified genes encoding glyceraldehyde-3-phosphate dehydrogenases (GAPCs) in glycolysis pathways that are up-regulated in drought tolerant lines, as reported in *Arabidopsis*. Glyceraldehyde-3-phosphate dehydrogenases may provide a direct connection between membrane lipid–based signaling, energy metabolism and growth control in a plant's response to ROS and water stress (Guo et al., 2012). Phosphoenol pyruvate carboxylase (PEPC) has been shown to participate in stress responses of C4 plants and C3 plants, as it served to balance carbon and nitrogen metabolism (Doubnerová and Ryšlavá, 2011). Despite the studies that have tried to understand the regulation and roles of PEPC, the role that this enzyme plays in drought stressed plants is unknown. PEPC might be involved in drought tolerance via alleviating the inhibitory effect of drought stress on photosynthesis (Bao-Yuan et al., 2011). In our study, up-regulation of PEPC could possibly improve drought tolerance by affecting the metabolism of glycolysis and gluconeogenesis. Additionally, other genes involved in the glycolysis and gluconeogenesis pathways were not previously reported to be associated with drought response. Therefore, further studies are needed to understand the mechanism modulating plant growth under stress.

Similarly, we investigated the effect of drought on the activities of DEGs associated with starch and sucrose metabolism. Trehalose 6-P phosphatase (TPP) was a key enzyme that significantly enhanced drought resistance in a variety of organisms (Pilon-Smits et al., 1998). Interestingly, transgenic plants that express TPP genes from microorganisms not only exhibit an increase in drought tolerance, but also show strong developmental alterations (Jang et al., 2003). In the current study, the up-regulation of TPP may have played a role in the response of resistant lines to drought, thus resulting in changes in carbohydrate allocation and metabolism (Redillas et al., 2012). Phosphoglucomutase (PGM) contributes to the coordination of sucrose synthesis with the rate of carbon dioxide fixation, and to the control of partitioning of photosynthate between sucrose and starch (Toldi et al., 2002; Nielsen et al., 2004). There was a slight decrease in PGM in drought-stressed wheat leaves (Zhu, 2001). However, we observed an up-regulation of PGM in the drought resistant lines of annual ryegrass, which agreed with the findings of Pinheiro et al. (2008). GPI has been shown to fill unique roles in carbohydrate metabolism during drought stress in different plants, e.g., *Arabidopsis* (Seki et al., 2002) and wheat (Xue et al., 2013). In seedling development under water stress, the decrease in the expression of starch phosphorylase might be due to inhibitory effects on starch concentrations (Peng et al., 2014). Compared with the drought sensitive lines, the drought tolerant line maintained higher activity of leaf starch phosphorylase in the chickpea (Awasthi et al., 2014). Inconsistent with our results, drought affected the activity of starch phosphorylase in annual ryegrass and showed up-regulated expression in the drought resistant lines. The change in the up-regulated expression of genes related to starch and sucrose metabolism after drought stress was striking, indicating that drought resistant annual ryegrass lines invests more energy and resources into immediate defense needs than the susceptible lines.

### Lipid metabolism-related genes and drought-induced genes used as antioxidants

Under drought conditions, considerable changes in lipid metabolism were observed (Benhassaine-Kesri et al., 2002). Previous studies have investigated responsive genes of lipid metabolism in ryegrass (Foito et al., 2013), but the results have not been previously linked with annual ryegrass's response to drought stress. We identified five related up-regulated genes in drought resistant lines under drought stress. Genes encoding glutathione reductase (GSR) and GGT were identified as important enzymes in glycerophospholipid metabolism. The down-regulation of the GGT gene might play a negative regulatory role in GSH degradation and the GSR gene may have been up-regulated when subjected to drought stress, which consequently mitigated the oxidative stress (Fan et al., 2014). In the present study, we observed up-regulation of genes encoding GSR and GGT, suggesting that the two enzymes at least partially contribute to the high drought tolerance in annual ryegrass. It has been widely reported that L-ascorbate peroxidase (AP) participated in the ascorbate-glutathione cycle, which is an important process for free radical detoxification (Cramer et al., 2013). Significant up-regulation of the gene encoding AP was identified in this study; therefore, AP activity may influence drought tolerance by regulating glycerophospholipid metabolism and the ascorbate pathway in drought resistant lines to adapt to water deficit. In plants, a6-phosphogluconate dehydrogenase (6-PGD) and G6PDH are major sources of NADPH in the cytoplasm of plant cells. These key enzymes are also used for the detoxification of hydrogen peroxide through the ascorbate-glutathione cycle. A previous study found that the activities of 6-PGD and G6PDH increased when plants were subjected to many artificial or transitory stressors, except in reed (*Phragmites communis*) under long-term drought stress (Chen et al., 2003). This conclusion holds true for short-term drought stress adaptation in annual ryegrass.

### Signal transduction-related genes and transcription factors associated with drought tolerance

Plants perceive environmental signals and transmit the signals to the cellular machinery to activate adaptive response in plants under abiotic stress (Xiong et al., 2002). Signal transduction requires certain molecules that participate in the modification, delivery, and assembly of signaling components (Ji et al., 2013). Genes encoding 1-phosphatidylinositol-4-phosphate 5-kinase (PIP kinase), which is involved in the defense response in poplars (Chen and Cao, 2015), plays a signaling role and the enzyme is a component of a signaling pathway associated with the synthesis of phosphatidylinositol phosphate (Gupta et al., 2013). Phosphatidylinositol-specific phospholipase C (PIase C) plays a central role in the phosphatidylinositol-specific signal transduction pathway. Georges et al. (2009) found that the over-expression of PIase C in canola enhances drought tolerance and promotes early flowering and maturation. Therefore, the up-regulation of PIase C may dramatically influence drought tolerance by regulating signal transduction in drought resistant annual ryegrass lines. 1D-myo-Inositol-tetrakisphosphate 5-kinase has different expression in drought resistant and drought susceptible lines, but the specific molecular function of the enzyme is not clear. Phosphatidic acid (PA) plays a pivotal role in the plant's response to environmental signals (Arisz et al., 2009). PA can also be generated by DGK, which is thought to act as a switch with two functional implications: the termination of diacylglycerol (DAG) signaling and the initiation of PA signaling (Frere and Di Paolo, 2009). DGK has been reported in several plant species including tobacco, wheat, tomato, and *Arabidopsis* (Ge et al., 2012). In our study, up-regulated DGK-encoding genes have been found in the drought resistant lines of annual ryegrass. In addition, phosphatidate cytidylyltransferase catalyzes the synthesis reaction of triglycerides, which are mainly found in cell membranes (Longmuir and Johnston, 1980). It is evident that the expression of genes coding phosphatidate cytidylyltransferase played an important role in the induction of drought resistance in citrus fruits (Ballester et al., 2011).

In our study, some of the DEGs encoding transcriptional factors were identified in the drought tolerant line. A previous study has demonstrated that AUX1in *Arabidopsis* encodes a high-affinity auxin influx carrier, and Arabidopsis AUX/LAX genes encode a family of auxin influx transporters that perform distinct developmental functions and have evolved distinct regulatory mechanisms (Péret et al., 2012). Furthermore, auxin influx results in enhanced resistance and the improvement of plant growth (Remy et al., 2013). Our results also give evidence to support this. Nonexpressorofpathogenesis_relatedgenes1 (NPR1) is known to be involved in salicylic acid (SA)-mediated suppression of the jasmonic acid (JA) signaling pathway (Caarls et al., 2015). NPR1 is a regulator of basal and systemic acquired resistance in plants (Shimobayashi et al., 2013). Srinivasan et al. (2009) reported that *Arabidopsis* NPR1 enhances oxidative stress tolerance in transgenic tobacco plants. These results may explain why the expression of NPR1 genes was implicated in the drought tolerance of annual ryegrass. Histidine-containing phosphotransfer proteins (HPTs) have already been identified in the cytokinin transduction pathway (Hwang et al., 2002), which was isolated in a study on plant response to osmotic stress, salt stress, and drought stress (Chefdor et al., 2006). The HPTs were shown to up-regulate under drought stress and may help drought resistant ryegrass to withstand environmental stress. The genes involved in the signal transduction pathway contributed to the increase of drought tolerance in annual ryegrass.

## Author contributions

LP collected data and wrote the manuscript. XZ and XM designed the project. MZ served as scientific advisors. JW, XM, and LH participated in technical editing of the manuscript. Other authors improved resolution of images.

### Conflict of interest statement

The authors declare that the research was conducted in the absence of any commercial or financial relationships that could be construed as a potential conflict of interest.

## Supplementary material

Supplementary Table 1 **List of partial RNA-seq-identified gens in *L. multiflorum***.

Supplementary Figure 1 **Hierarchical clustering of differentially expressed genes**.

Supplementary Figure 2 **Contents of glucose (A) and fructose (B) in two *L. multiflorum* lines subjected to drought stress for 1 and 2 h**. Each value represents the mean ±SE (*n* = 4). The different letters indicate significant differences (*P* < 0.05) between different time points.
