# Supplementary material for: Transcriptional Profiles of Drought-Related Genes in Modulating Metabolic Processes and Antioxidant Defenses in Lolium multiflorum
Source: Front Plant Sci. 2016 Apr 25;7:519. doi: 10.3389/fpls.2016.00519 (PMC4842912; doi:10.3389/fpls.2016.00519)
Supplement: Supplementary Table 1 — List of partial RNA-seq-identified gens in L. multiflorum. [file Table1.DOCX]

**Supplementary Table 1 List of partial RNA-seq-identified gens in *L. multiflorum***

| **Accession.** | **Description** | **P value** | **Sources** |
| --- | --- | --- | --- |
| gi\|326497227 | Phosphoenolpyruvate carboxykinase (ATP) [EC:4.1.1.49] | 2.78E-18 | Hordeum vulgare subsp. vulgare |
| gi\|326491633 | isocitrate lyase [EC:4.1.3.1] | 3.97E-25 | Hordeum vulgare subsp. vulgare |
| gi\|298707515 | S-adenosylmethionine synthetase [EC:2.5.1.6] | 3.86E-11 | Ectocarpus siliculosus |
| gi\|226503259 | 12-oxophytodienoic acid reductase [EC:1.3.1.42] | 1.52E-35 | Zea mays |
| gi\|326491633 | isocitrate lyase [EC:4.1.3.1] | 1.94E-61 | Hordeum vulgare subsp. vulgare |
| gi\|225685855 | S-adenosylmethionine synthetase [EC:2.5.1.6] | 2.78E-43 | Ditylum brightwellii |
| gi\|326502814 | acetyl-CoA synthetase [EC:6.2.1.1] | 4.07E-89 | Hordeum vulgare subsp. vulgare |
| gi\|226503259 | 12-oxophytodienoic acid reductase [EC:1.3.1.42] | 4.43E-31 | Zea mays |
| gi\|125596002 | phospholipase D [EC:3.1.4.4] | 7.87E-25 | Oryza sativa Japonica Group |
| gi\|255597848 | NADPH2:quinone reductase [EC:1.6.5.5] | 2.00E-25 | Ricinus communis |
| gi\|302851281 | phospholipase D [EC:3.1.4.4] | 5.22E-15 | Volvox carteri f. nagariensis |
| gi\|299471880 | F-type H+-transporting ATPase subunit alpha [EC:3.6.3.14] | 6.68E-16 | Ectocarpus siliculosus |
| gi\|302853993 | malate synthase [EC:2.3.3.9] | 1.19E-09 | Volvox carteri f. nagariensis |
| gi\|323456170 | adenosylhomocysteinase [EC:3.3.1.1] | 5.22E-15 | Aureococcus anophagefferens |
| gi\|11465901 | cytochrome c oxidase subunit 1 [EC:1.9.3.1] | 5.51E-18 | Ochromonas danica |
| gi\|159475042 | malate synthase [EC:2.3.3.9] | 9.28E-09 | Chlamydomonas reinhardtii |
| gi\|354549249 | S-adenosylmethionine synthetase [EC:2.5.1.6] | 3.66E-08 | Nicotiana tabacum |
| gi\|452825047 | adenosylhomocysteinase [EC:3.3.1.1] | 9.81E-12 | Galdieria sulphuraria |
| gi\|357133814 | phospholipase D [EC:3.1.4.4] | 6.68E-16 | Brachypodium distachyon |
| gi\|326515786 | citrate synthase [EC:2.3.3.1] | 1.04E-14 | Hordeum vulgare subsp. vulgare |
| gi\|242064816 | DNA-directed RNA polymerase II [EC:2.7.7.6] | 4.94E-12 | Sorghum bicolor |
| gi\|159475042 | malate synthase [EC:2.3.3.9] | 8.79E-06 | Chlamydomonas reinhardtii |
| gi\|348686055 | isocitrate dehydrogenase [EC:1.1.1.42] | 9.81E-12 | Phytophthora sojae |
| gi\|356507105 | pectinesterase [EC:3.1.1.11] | 9.28E-09 | Glycine max |
| gi\|306415487 | delta8-fatty-acid desaturase [EC:1.14.19.4] | 9.81E-12 | Nannochloropsis oculata |
| gi\|460376065 | mitochondrial trans-2-enoyl-CoA reductase [EC:1.3.1.38] | 9.28E-09 | Solanum lycopersicum |
| gi\|224136808 | cathepsin L [EC:3.4.22.15] | 4.68E-09 | Populus trichocarpa |
| gi\|298715508 | adenosylhomocysteinase [EC:3.3.1.1] | 3.19E-13 | Ectocarpus siliculosus |
| gi\|255548784 | chitinase [EC:3.2.1.14] | 6.87E-05 | Ricinus communis |
| gi\|414888148 | peptidyl-prolyl isomerase G (cyclophilin G) [EC:5.2.1.8] | 1.25E-12 | Zea mays |
| gi\|168013337 | citrate synthase [EC:2.3.3.1] | 4.43E-06 | Physcomitrella patens subsp. patens |
| gi\|348690313 | ATP-dependent RNA helicase [EC:3.6.4.13] | 5.99E-10 | Phytophthora sojae |
| gi\|428184211 | aldehyde dehydrogenase (NAD+) [EC:1.2.1.3] | 8.09E-14 | Guillardia theta CCMP2712 |
| gi\|303290340 | 2-methylcitrate dehydratase [EC:4.2.1.79] | 5.67E-07 | Micromonas pusilla CCMP1545 |
| gi\|300681556 | (S)-beta-macrocarpene synthase [EC:5.5.1.17] | 3.66E-08 | Triticum aestivum |
| gi\|299116858 | delta8-fatty-acid desaturase [EC:1.14.19.4] | 4.68E-09 | Ectocarpus siliculosus |
| gi\|326515786 | citrate synthase [EC:2.3.3.1] | 4.08E-14 | Hordeum vulgare subsp. vulgare |
| gi\|326497939 | malate dehydrogenase (decarboxylating) [EC:1.1.1.39] | 1.74E-05 | Hordeum vulgare subsp. vulgare |
| gi\|302828408 | phospholipase D [EC:3.1.4.4] | 6.32E-13 | Volvox carteri f. nagariensis |
| gi\|159487004 | pectinesterase [EC:3.1.1.11] | 1.74E-05 | Chlamydomonas reinhardtii |
| gi\|324983196 | cytochrome c oxidase subunit 1 [EC:1.9.3.1] | 4.43E-06 | Halophytophthora mycoparasitica |
| gi\|384245617 | pyruvate decarboxylase [EC:4.1.1.1] | 1.79E-19 | Coccomyxa subellipsoidea C-169 |
| gi\|299471123 | cathepsin B [EC:3.4.22.1] | 1.95E-11 | Ectocarpus siliculosus |
| gi\|293335365 | mannitol-1-phosphate 5-dehydrogenase [EC:1.1.1.17] | 4.08E-14 | Zea mays |
| gi\|255593202 | propionyl-CoA synthetase [EC:6.2.1.17] | 3.66E-08 | Ricinus communis |
| gi\|224005467 | F-type H+-transporting ATPase subunit alpha [EC:3.6.3.14] | 1.61E-13 | Thalassiosira pseudonana CCMP1335 |
| gi\|302851281 | pectinesterase [EC:3.1.1.11] | 2.09E-60 | Volvox carteri f. nagariensis |
| gi\|460406243 | phospholipase D [EC:3.1.4.4] | 5.25E-40 | Solanum lycopersicum |
| gi\|388492304 | adenosylhomocysteinase [EC:3.3.1.1] | 8.25E-95 | Lotus japonicus |
| gi\|260516654 | cathepsin L [EC:3.4.22.15] | 2.28E-78 | Brachiaria hybrid cultivar |
| gi\|348682503 | ATP-dependent RNA helicase DDX46/PRP5 [EC:3.6.4.13] | 1.38E-79 | Phytophthora sojae |
| gi\|428169555 | S-adenosylmethionine synthetase [EC:2.5.1.6] | 6.29E-62 | Guillardia theta CCMP2712 |
| gi\|388514675 | glyceraldehyde 3-phosphate dehydrogenase [EC:1.2.1.12] | 2.16E-25 | Lotus japonicus |
| gi\|159484660 | peptidylprolyl isomerase [EC:5.2.1.8] | 2.19E-11 | Chlamydomonas reinhardtii |
| gi\|242058141 | phospholipase D [EC:3.1.4.4] | 2.15E-32 | Sorghum bicolor |
| gi\|302828406 | phospholipase D [EC:3.1.4.4] | 4.36E-25 | Volvox carteri f. nagariensis |
| gi\|255079428 | FK506-binding nuclear protein [EC:5.2.1.8] | 7.93E-28 | Micromonas sp. RCC299 |
| gi\|347979000 | peptidylprolyl isomerase [EC:5.2.1.8] | 9.80E-15 | Plasmopara viticola |
| gi\|302768691 | phosphoglycolate phosphatase [EC:3.1.3.18] | 8.67E-39 | Selaginella moellendorffii |
| gi\|388491104 | nucleoside-diphosphate kinase [EC:2.7.4.6] | 8.84E-18 | Lotus japonicus |
| gi\|326503078 | glutathione S-transferase [EC:2.5.1.18] | 1.78E-17 | Hordeum vulgare subsp. vulgare |
| gi\|168067009 | phospholipase D [EC:3.1.4.4] | 7.20E-24 | Physcomitrella patens subsp. patens |
| gi\|308044587 | formate dehydrogenase [EC:1.2.1.2] | 4.39E-18 | Zea mays |
| gi\|326503078 | glutathione S-transferase [EC:2.5.1.18] | 2.66E-19 | Hordeum vulgare subsp. vulgare |
| gi\|302834718 | F-type H+-transporting ATPase subunit beta [EC:3.6.3.14] | 1.95E-28 | Volvox carteri f. nagariensis |
| gi\|326503984 | cathepsin L [EC:3.4.22.15] | 3.20E-34 | Hordeum vulgare subsp. vulgare |
| gi\|449019164 | 3-oxoacyl-[acyl-carrier protein] reductase [EC:1.1.1.100] | 8.78E-25 | Cyanidioschyzon merolae strain 10D |
| gi\|159466892 | F-type H+-transporting ATPase subunit beta [EC:3.6.3.14] | 1.09E-11 | Chlamydomonas reinhardtii |
| gi\|168004121 | nucleoside-diphosphate kinase [EC:2.7.4.6] | 1.47E-09 | Physcomitrella patens subsp. Patens |
| gi\|115479041 | cinnamyl-alcohol dehydrogenase [EC:1.1.1.195] | 2.43E-08 | Oryza sativa Japonica Group |
| gi\|449018731 | H+-transporting ATPase [EC:3.6.3.6] | 7.20E-24 | Cyanidioschyzon merolae strain 10D |
| gi\|326508398 | glutamate dehydrogenase (NAD(P)+) [EC:1.4.1.3] | 1.20E-08 | Hordeum vulgare subsp. vulgare |
| gi\|224006397 | FK506-binding protein 1 [EC:5.2.1.8] | 7.29E-10 | Thalassiosira pseudonana CCMP1335 |
| gi\|384251171 | glycine hydroxymethyltransferase [EC:2.1.2.1] | 1.63E-06 | Coccomyxa subellipsoidea C-169 |
| gi\|301119899 | fructose-bisphosphate aldolase, class II [EC:4.1.2.13] | 4.83E-22 | Phytophthora infestans T30-4 |
| gi\|326521524 | peptidylprolyl isomerase [EC:5.2.1.8] | 9.80E-15 | Hordeum vulgare subsp. vulgare |
| gi\|301107640 | cinnamyl-alcohol dehydrogenase [EC:1.1.1.195] | 1.96E-21 | Phytophthora infestans T30-4 |
| gi\|325187599 | cinnamyl-alcohol dehydrogenase [EC:1.1.1.195] | 2.64E-26 | Albugo laibachii Nc14 |
| gi\|325180734 | fructose-bisphosphate aldolase, class II [EC:4.1.2.13] | 5.97E-09 | Albugo laibachii Nc14 |
| gi\|302850517 | phospholipase D [EC:3.1.4.4] | 3.61E-10 | Volvox carteri f. nagariensis |
| gi\|115453823 | pectinesterase [EC:3.1.1.11] | 5.97E-09 | Oryza sativa Japonica Group |
| gi\|326499079 | F-type H+-transporting ATPase subunit beta [EC:3.6.3.14] | 1.63E-06 | Hordeum vulgare subsp. vulgare |
| gi\|357138111 | L-ascorbate oxidase [EC:1.10.3.3] | 2.61E-19 | Brachypodium distachyon |
| gi\|357143439 | calcium-dependent protein kinase [EC:2.7.11.1] | 4.16E-07 | Brachypodium distachyon |
| gi\|326490451 | trans-resveratrol di-O-methyltransferase [EC:2.1.1.240] | 1.88E-61 | Hordeum vulgare subsp. Vulgare |
| gi\|326525010 | lysophospholipase III [EC:3.1.1.5] | 8.82E-15 | Hordeum vulgare subsp. Vulgare |
| gi\|326487618 | phospholipase D [EC:3.1.4.4] | 1.88E-17 | Hordeum vulgare subsp. Vulgare |
| gi\|384247726 | cytochrome-b5 reductase [EC:1.6.2.2] | 5.98E-06 | Coccomyxa subellipsoidea C-169 |
| gi\|357158487 | S-adenosylmethionine decarboxylase [EC:4.1.1.50] | 8.37E-34 | Brachypodium distachyon |
| gi\|357161517 | cysteamine dioxygenase [EC:1.13.11.19] | 2.60E-89 | Brachypodium distachyon |
| gi\|242084592 | phospholipase D [EC:3.1.4.4] | 1.96E-08 | Sorghum bicolor |
| gi\|115467912 | xyloglucan:xyloglucosyl transferase [EC:2.4.1.207] | 1.96E-08 | Oryza sativa Japonica Group |
| gi\|326525363 | tRNA (cytosine38-C5)-methyltransferase [EC:2.1.1.204] | 8.76E-05 | Hordeum vulgare subsp. vulgare |
