# Supplementary figures and images for: Transcriptional Profiles of Drought-Related Genes in Modulating Metabolic Processes and Antioxidant Defenses in Lolium multiflorum
Source: Front Plant Sci. 2016 Apr 25;7:519. doi: 10.3389/fpls.2016.00519 (PMC4842912; doi:10.3389/fpls.2016.00519)

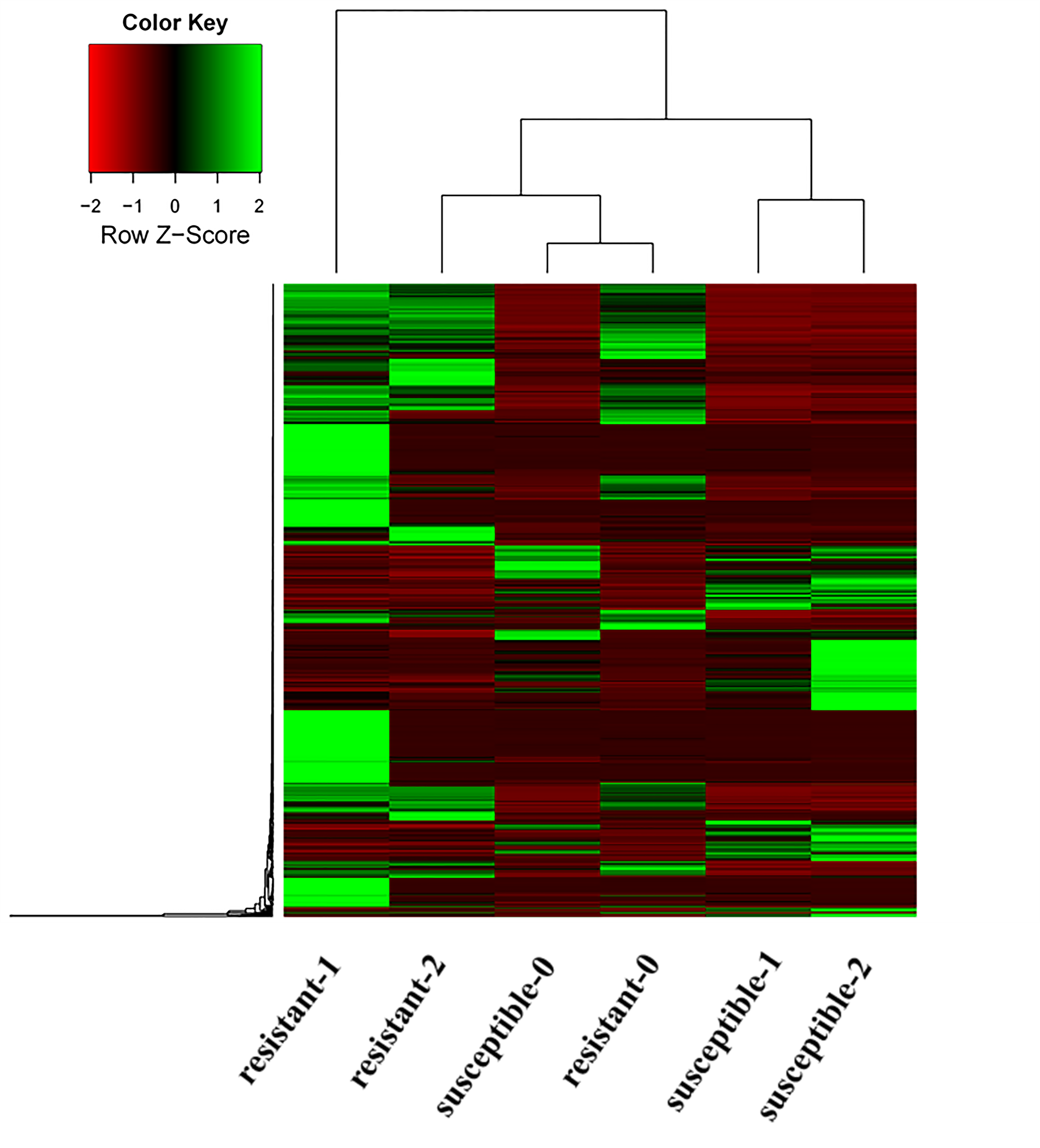

Supplement: Supplementary Figure 1 — Hierarchical clustering of differentially expressed genes. [file Image1.TIF]

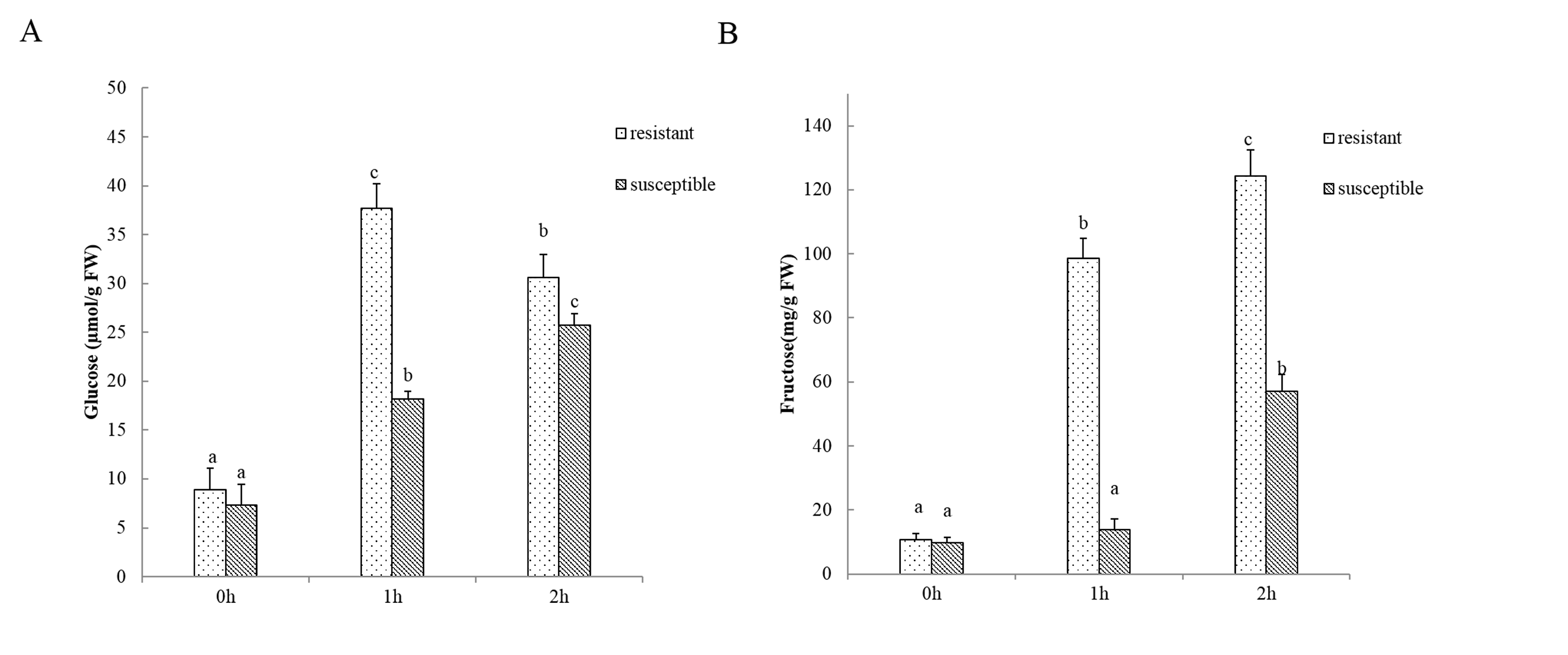

Supplement: Supplementary Figure 2 — Contents of glucose (A) and fructose (B) in two L. multiflorum lines subjected to drought stress for 1 and 2 h. Each value represents the mean ±SE (n = 4). The different letters indicate significant differences (P < 0.05) between different time points. [file Image2.TIF]
